# Risk factors of nasogastric tube removal failure in acute ischemic stroke patients: a 3-months follow-up cohort study at a single institute

**DOI:** 10.3389/fneur.2026.1801145

**Published:** 2026-07-01

**Authors:** Ya-Chi Chuang, Hsuan-Mei Huang, Po-Lin Chen, Jin-An Huang, Yuan-Yang Cheng, Jimmy Chun-Ming Fu

**Affiliations:** 1 Department of Physical Medicine and Rehabilitation, Taichung Veterans General Hospital, Taichung, Taiwan; 2 Department of Industrial Engineering and Enterprise Information, Tunghai University, Taichung, Taiwan; 3 Division of Neurology, Neurological Institute, Taichung Veterans General Hospital, Taichung, Taiwan; 4 School of Medicine, National Yang Ming Chiao Tung University, Taipei, Taiwan; 5 Department of Post-Baccalaureate Medicine, College of Medicine, National Chung Hsing University, Taichung, Taiwan; 6 Department of Medical Administration, Taichung Veterans General Hospital, Taichung, Taiwan

**Keywords:** dysphagia, ischemic strokes, nasogastric tube, risk factors, speech therapy

## Abstract

**Background:**

Post-stroke dysphagia is an important problem affecting patient outcomes, and frequently necessitating immediate nasogastric tube (NGT) placement for enteral nutrition. While initially recommended, the prolonged use of NGT leads to more complications. The study aims to identify risk factors for failure of NGT removal at discharge and 3 months post-discharge in acute ischemic stroke patients.

**Methods:**

A retrospective analysis of 290 patients admitted from January 2022 to November 2023 was performed in a single center. All included patients strictly underwent this standardized dysphagia evaluation protocol before any NGT placement or removal decisions were made, ensuring the clinical validity of our outcome measures. Data on demographics, clinical characteristics, stroke-related factors (stroke severity, location, etiology, and functional status) and rehabilitation-related factors (post-discharge rehabilitation setting) were extracted and analyzed using logistic regression.

**Results:**

Several factors, including age, pre-existing comorbidities, modified Rankin Scale score, stroke location, stroke etiology (cardioembolic type), severity, consciousness level, hand and lower limb Brunnstrom stage, intensive care unit stay length, post-discharge rehabilitation setting and number of speech therapy sessions during admission, were statistically significant risk factors for NGT removal failure at 3 months post-discharge. Multivariate analysis later revealed from these items, only the cardioembolic stroke (OR 10.31, *p* = 0.024) remained the most significant independent predictor. However, the exceptionally wide confidence interval (95% CI 1.35–78.63) suggests limited precision and possible model instability due to sparse data within that specific subcategory; therefore, this finding should be interpreted cautiously.

**Conclusion:**

These preliminary findings suggest an association between functional status, stroke etiology, and NGT removal outcomes. Given the retrospective exploratory nature of this study, further prospective research is needed to establish definitive causal relationships.

## 1 Introduction

Stroke is a leading cause of death and disability worldwide. Ischemic stroke constitutes up to 80% of all stroke cases and poses substantial economic and societal burdens (1, 2). Malnutrition, often caused by dysphagia, is linked to poor stroke outcomes, resulting in feeding through a nasogastric tube (NGT) (3, 4). A recent meta-analysis reported a 42% pooled prevalence of post-stroke dysphagia (5). The American Heart Association/American Stroke Association 2019 guidelines for acute ischemic stroke management also recommend the initial NGT use in feeding dysphagic patients (6).

However, the prolonged use of NGTs is associated with multiple complications, including nasal wing lesion, chronic sinusitis, gastro-esophageal reflux, aspiration pneumonia and even impaired swallowing functions (7, 8). Also, the use of NGT likely presents cosmetic issues, interfering with rehabilitation process, and impairing the overall quality of life. Thus, recognizing the factors that influence the successful removal of NGTs is crucial for improving rehabilitation outcomes.

Previous studies have identified several factors influencing the successful removal of NGT upon discharge in stroke patients, including age, functional independence, language comprehension ability (9, 10), and findings from video fluoroscopic swallowing studies (11). Few studies have investigated other stroke-related factors, including severity, etiology, location, and motor recovery stages as well as rehabilitation-related factors. Previous research mainly focused on NGT status at hospital discharge and no studies have investigated at longer timeframes. Given that stroke recovery is a relatively lengthy process and recent literature indicates that the first 2–3 months post-stroke represent a critical window of heightened neuroplasticity for motor and functional recovery (12). Examining changes over 3-months periods could provide deeper and more meaningful insights. Here, we aimed to study risk factors of the failure of nasogastric tube removal both at hospital discharge and at 3-months post-discharge. Furthermore, the present study provides a novel contribution by integrating multidimensional stroke-specific variables—such as precise TOAST etiology, lesion location, Brunnstrom motor recovery stages, and post-discharge rehabilitation settings—into a longitudinal predictive model.

## 2 Methods

### 2.1 Participants and swallowing assessment protocol

A retrospective study was conducted to identify acute ischemic stroke patients who received NGT feeding during hospitalization at our hospital during the period January 2022 to November 2023. During hospitalization, patients underwent a highly standardized clinical swallowing evaluation protocol. Nurses routinely performed non-instrumental dysphagia screening using the Yale Swallow Protocol. Patients who failed the screening or exhibited severe neurological deficits received NGT placement for safety and were rapidly referred to rehabilitation doctor and speech therapist. Standardized speech therapy interventions included oral motor exercises, sensory stimulation, and compensatory postural strategies. The decision for NGT removal was never arbitrary; it was made collaboratively by physicians and speech therapist based on progressive clinical improvements. When bedside clinical assessments were equivocal regarding aspiration risk, objective instrumental assessments, such as Videofluoroscopic Swallowing Studies (VFSS), were routinely utilized to guide the final removal decision. Patients were recruited if they aged 18 years or older. We excluded those patients who had died during admission or received NGT feeding prior to the current episode of stroke. This study received ethical board approval by our institution (No. CE24339C).

### 2.2 Data extraction

Retrospective data on demographics and clinical information were first extracted, including sex, age, body mass index (BMI), Glasgow Coma Scale (GCS) score at admission, length of stay at intensive care unit (ICU), and pre-existing comorbidities. In addition, we recorded their stroke-related variables such as etiology, severity [measured by initial National Institute of Health Stroke Scale, (NIHSS) score], location, Brunnstrom stage as well as functional status at discharge (measured by the modified Rankin Scale, mRS). The categorization of functional scales was clinically justified to prevent statistical noise while maintaining interpretability. The mRS scores were grouped into 1–2 (functionally independent or slight disability), 3–4 (moderate to moderately severe disability requiring assistance), and 5 (severe disability/bedridden); this precisely aligns with Taiwan’s strict Post-Acute Care (PAC) enrollment criteria, which require an mRS of 2–4. Brunnstrom stages for motor recovery were dichotomized into stages 1–2 (flaccidity to minimal spasticity) and stages 3–5 (emergence of voluntary movement). This cutoff represents a crucial physiological milestone, as basic voluntary motor control is an absolute prerequisite for a patient to actively maintain sitting balance and participate in swallowing rehabilitation. The etiology of stroke was determined using the TOAST classification, which categorizes strokes into five types: (1) large-artery atherosclerosis (LAA), (2) cardioembolism, (3) small-vessel occlusion, (4) stroke of other determined etiology, and (5) stroke of undetermined etiology. Experienced physicians determined the ischemic stroke locations based on CT or MRI scans, following standard clinical protocols (13). The location included four subtypes: (1) cortical stroke involving one hemisphere, (2) subcortical stroke involving one hemisphere, (3) brainstem and/or cerebellum stroke (not multifocal), and (4) mixed or multifocal stroke. Brunnstrom stages tested at hospital discharge were categorized into 2 groups: stages 1–2 (no voluntary movement) and stages 3–5 (having voluntary movement). The mRS scores were grouped into three categories: 1–2, 3–4, and 5, based on the Post-Acute Care (PAC) enrollment criteria. Moreover, rehabilitation-related factors, including the post-discharge rehabilitation setting and the number of speech therapy sessions during admission, were also extracted. Post-discharge rehabilitation settings were divided into four types: home, PAC inpatient rehabilitation, standard inpatient rehabilitative care, and nursing facilities. The Post-acute Care-Cerebrovascular Diseases Project was launched in Taiwan in 2014, utilizing a multidisciplinary team in regional/district hospitals to reduce healthcare costs and achieve equal or even better patient outcomes. The major difference between PAC and standard inpatient rehabilitative care lies in that PAC provides greater intensity of rehabilitation, with stricter criteria on patient inclusion, for those with moderate to severe functional disability (mRS 3–4) (14). Patients were able to choose freely their post-discharge rehabilitation settings through a Shared Decision-Making process.

### 2.3 Statistical analyses

For the descriptive analysis, continuous data are reported as mean and standard deviation while categorical data are shown as percentage. The normal distribution of continuous data was checked with the Kolmogorov–Smirnov test. We used two sample *t*-tests, Mann–Whitney *U* test or chi-square test to compare baseline variables between the group with successful NGT removal 3-months after discharge, and the group with failed NGT removal. Logistic regression was conducted to identify risk factors for the failure of NGT removals. If the events per variable in the logistic regression was less than 10, the variable was removed to avoid bias (15). Overall model performance was rigorously assessed: model calibration was evaluated using the Hosmer–Lemeshow goodness-of-fit test (where *p* > 0.05 indicates an adequate fit), and model discrimination was quantified using the C-statistic (Area Under the Receiver Operating Characteristic curve, AUC). To ensure the stability of the multivariable logistic regression models, multicollinearity diagnostics were strictly performed prior to model construction by calculating the Variance Inflation Factor (VIF) and Tolerance. Variables with VIF > 5.0 or Tolerance < 0.20 were considered to have severe collinearity and were scrutinized. Highly interrelated clinical severity indicators (such as GCS, NIHSS, and ICU stay) were selectively managed to prevent standard error inflation.

Statistical analyses were performed with IBM SPSS Statistics software (SPSS Inc., Chicago, IL, United States, v.23). Significant level was set at *p* < 0.05.

## 3 Results

### 3.1 Demographic and clinical characteristics of patients

**Figure 1:**
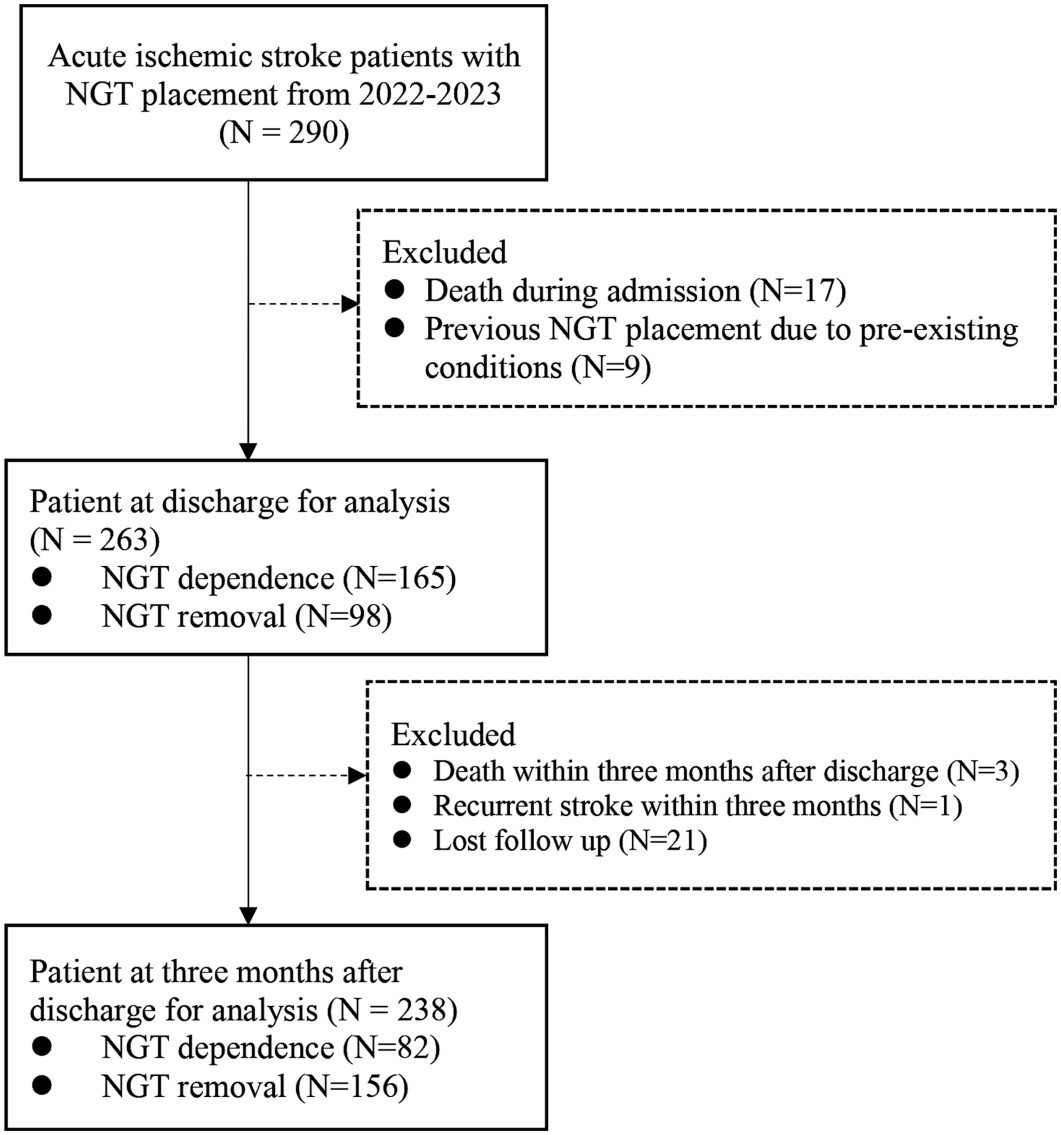
The flow diagram of the study design.

Between January 2022 and November 2023, we screened 290 adult acute ischemic stroke patients with NGT feeding for eligibility of our study. Among them, 26 patients were excluded due to death during admission or previous NGT placement. After that, 264 patients were then included in the analysis at hospital discharge. Another 26 patients were later excluded due to death within 3-months post-discharge, recurrent stroke or lost in follow-up. Finally, 238 patients were in the analysis at 3-months after discharge. Details of patient enrollment are shown in Figure 1. The demographics and descriptive data for the group with NGT removal 3-months after discharge, and the group with failed NGT removal are both shown in Table 1. We found significant differences between two groups in terms of the following, age, pre-existing atrial fibrillation (Af), diabetes mellitus (DM), cerebrovascular accident (CVA) history, mRS score, lesion location, NIHSS, GCS level, hand and lower limb Brunnstrom stage, ICU stay length, post-discharge rehabilitation setting and number of ST sessions during admission.

**Table 1:** Patient characteristics.

Variables	NG tube removed at 3 months (*N* = 156)	Failure of NG tube removal at 3 months (*N* = 82)	*p*-value
Sex (male)	100 (64.1%)	46 (56.1%)	0.228
Age	68.4 ± 12.88	74.1 ± 10.42	**0.001** ^*^
BMI	24.74 ± 3.66	23.82 ± 3.59	0.087
HTN	116 (74.4%)	70 (85.4%)	0.051
Atrial fibrillation	61 (39.1%)	47 (57.3%)	**0.007** ^*^
DM	54 (34.6%)	47 (57.3%)	**0.001** ^*^
CVA history	21 (13.5%)	21 (25.6%)	**0.019** ^*^
Barthel index			
mRS			**<0.001** ^*^
1, 2	22 (14.3%)	0(0.0%)	
3, 4	105 (68.2%)	30(37.0%)	
5	27 (17.5%)	51(63.0%)	
Lesion location			**0.005** ^*^
Cortical stroke involving one hemisphere	86 (55.1%)	56(68.3%)	
Subcortical stroke involving one hemisphere	24 (15.4%)	5(6.1%)	
Brainstem + cerebellum stroke (not multifocal)	23 (14.7%)	3(3.7%)	
Mixed/multifocal stroke	23 (14.7%)	18(22.0%)	
TOAST classification			0.118
LAA	38 (29.2%)	13(18.8%)	
Cardioembolic	68 (52.3%)	49(71.0%)	
Small vessel occlusion	5 (3.8%)	2(2.9%)	
Other determine	4 (3.1%)	0 (0.0%)	
Undetermined	15 (11.5%)	5 (7.2%)	
NIHSS	14 ± 6.77	18.44 ± 6.68	**<0.001** ^*^
GCS ≥ 14	62 (39.7%)	17 (20.7%)	**0.003** ^*^
Hand Brunnstrom (ref: I/II)	53 (72.6%)	15 (31.3%)	**<0.001** ^*^
Lower limb Brunnstrom (ref: I/II)	58 (79.5%)	17 (35.4%)	**<0.001** ^*^
ICU stay length	3.34 ± 4.02	6.89 ± 9.09	**0.001** ^*^
Post-discharge rehabilitation setting			**<0.001** ^*^
Home	45 (28.8%)	13 (15.9%)	
Standard rehabilitative care	45 (28.8%)	45 (54.9%)	
PAC	63 (40.4%)	15 (18.3%)	
Nursing facility	3 (1.9%)	9 (11.0%)	
Number of ST sessions during admission	1.17 ± 1.23	0.65 ± 0.81	**0.001** ^*^

BMI, body mass index; HTN, hypertension; DM, diabetes mellitus; CVA, cerebrovascular accident; mRS, modified Rankin Scale; TOAST, Trial of ORG 10172 in Acute Stroke Treatment; LAA, large-artery atherosclerosis; NIHSS, National Institute of Health Stroke Scale; GCS, Glasgow Coma Scale; ref, reference; ICU, intensive care unit; PAC, Post-Acute Care; ST, speech therapy. *indicates statistical significance (*p* < 0.05).

### 3.2 Risk factors for failure of NGT removal at 3 months after discharge

Univariate and multivariate logistic regressions were performed to determine risk factors. Results are shown in Table 2 in terms of regression coefficient [95% confidence interval (CI)] and *p*-value. To prevent mathematical non-convergence and model instability, subgroups exhibiting absolute zero-cell counts (causing complete separation) were excluded from the multivariable logistic regression (15). Specifically, at 3 months post-discharge, this included patients with baseline mRS 1–2 (*n* = 22, all of whom achieved successful NGT removal, creating a perfect separation with zero failures), and the TOAST category of other determined etiology (*n* = 4, with zero failures). Additionally, rare etiological subgroups with very low cell counts (small-vessel occlusion, *n* = 7; undetermined etiology, *n* = 20) were excluded to prevent sparse data bias. While this exclusion prevents statistical inflation and standard error explosion, it limits our ability to mathematically estimate the independent odds ratios for these low-prevalence subgroups. Despite several factors were statistically significant risk factors for NGT removal failure in univariate logistic regression [i.e., age, pre-existing hypertension, DM, Af, mRS score, lesion location, TOAST classification (cardioembolic type), NIHSS, GCS level<14, hand and lower limb Brunnstrom stage, ICU stay length, post-discharge rehabilitation setting and number of ST sessions during admission], multivariate analyses revealed only the cardioembolic stroke (OR 10.31, 95% CI 1.35–78.63, *p* = 0.024) was the most significant independent predictor.

**Table 2:** Univariate and multivariate logistic analyses of risk factors for failure of nasogastric tube removal 3 months after discharge.

Variables	Univariate analysis		Multivariate analysis	
	OR (95%CI)	*p*-value	OR (95%CI)	*p*-value
Sex (ref:male)	1.28 (0.71–2.32)	0.416		
Age	1.04 (1.01–1.07)	**0.004** ^*^	1.03 (0.97–1.09)	0.359
BMI	0.95 (0.87–1.04)	0.262		
HTN	2.13 (1.02–4.47)	**0.045** ^*^	0.31 (0.03–3.54)	0.347
Atrial fibrillation	2.01 (1.11–3.63)	**0.021** ^*^	0.24 (0.04–1.58)	0.137
DM	2.14 (1.18–3.87)	**0.012** ^*^	2.55 (0.46–14.19)	0.285
CVA history	1.73 (0.81–3.68)	0.156		
mRS				
3, 4 (control)	1.00	**–**		
5	6.35 (3.30–12.23)	**<0.001** ^*^	4.53 (0.60–34.34)	0.144
Lesion location				
Cortical stroke involving one hemisphere (control)	1.00	–		
Subcortical stroke involving one hemisphere	0.27 (0.09–0.85)	**0.025** ^*^	0.70 (0.03–14.53)	0.820
Brainstem + cerebellum stroke (not multifocal)	0.15 (0.03–0.70)	**0.015** ^*^	4.03 (0.16–102.72)	0.399
Mixed/multifocal stroke	1.01 (0.46–2.21)	0.979	1.10 (0.11–11.49)	0.935
TOAST classification				
LAA (control)	1.00	–		
Cardioembolic	2.54 (1.21–5.35)	**0.014** ^*^	10.31 (1.35–78.63)	**0.024** ^*^
NIHSS	1.11 (1.05–1.16)	**<0.001** ^*^	1.14 (0.98–1.32)	0.081
GCS ≥ 14	0.38 (0.20–0.75)	**0.005** ^*^	0.18 (0.02–1.42)	0.103
Hand Brunnstrom (ref: I/II)	0.18 (0.08–0.43)	**<0.001** ^*^	0.58 (0.03–9.78)	0.704
Lower limb Brunnstrom (ref: I/II)	0.15 (0.06–0.37)	**<0.001** ^*^	1.07 (0.07–17.53)	0.962
ICU stay length	1.09 (1.03–1.15)	**0.002** ^*^	1.03 (0.88–1.20)	0.752
Post-discharge rehabilitation setting				
Home (control)	1.00	–		
Standard rehabilitative care	2.78 (1.21–6.40)	**0.016** ^*^	2.11 (0.24–18.46)	0.500
PAC	0.62 (0.24–1.57)	0.310	0.21 (0.02–2.67)	0.228
Nursing facility	6.06 (1.35–27.28)	**0.019** ^*^	1.64 (0.11–23.92)	0.719
Number of ST sessions during admission	0.61 (0.44–0.84)	**0.003** ^*^	0.98 (0.42–2.29)	0.957

OR, odds ratio; CI, confidence interval; ref, reference; BMI, body mass index; HTN, hypertension; DM, diabetes mellitus; CVA, cerebrovascular accident; mRS, modified Rankin Scale; TOAST, Trial of ORG 10172 in Acute Stroke Treatment; LAA, large-artery atherosclerosis; NIHSS, National Institute of Health Stroke Scale; GCS, Glasgow Coma Scale; ICU, intensive care unit; PAC, Post-Acute Care; ST, speech therapy Model performance indices demonstrated satisfactory fit: Hosmer–Lemeshow test *p* > 0.05; C-statistic AUC > 0.75. *indicates statistical significance (*p* < 0.05). Bold values indicate statistically significant independent variables in the multivariate logistic regression model (*p* < 0.05).

### 3.3 Risk factors for failure of NGT removal at discharge

If we only considered the risk factors for patients who still had an NGT at discharge, logistic regression was done. Due to the insufficient number of patients with mRS scores of 1 and 2, we excluded those patients (Supplementary Table S1, showing only 2 patients with NGT at discharge). This complete NGT removal among mRS 1–2 patients at discharge created a perfect separation (zero failures), preventing their inclusion in the multivariable model and highlighting mild baseline disability as a strong clinical, though unmodellable, predictor of early NGT weaning. Several factors revealed significantly larger odds ratios in the univariate logistic regression analysis. They were age, BMI, pre-existing Af, mRS score, NIHSS, GCS level, hand and lower limb Brunnstrom stage, ICU stay length and number of ST sessions during admission. However, in the multivariate logistic regression, we found no variables that were statistically significant predictors of NGT removal at discharge. Detailed results are shown in Table 3.

**Table 3:** Univariate and multivariate logistic analyses of risk factors for failure of nasogastric tube removal after discharge.

Variables	Univariate analysis		Multivariate analysis	
	OR (95%CI)	*p*-value	OR (95%CI)	*p*-value
Sex (ref: male)	1.09 (0.63–1.89)	0.749		
Age	1.03 (1.01–1.06)	**0.003** ^*^	1.04 (1.00–1.08)	0.065
BMI	0.90 (0.83–0.98)	**0.011** ^*^	0.90 (0.79–1.03)	0.117
HTN	0.90 (0.47–1.75)	0.763		
Atrial fibrillation	1.82 (1.05–3.16)	**0.034** ^*^	1.36 (0.43–4.36)	0.601
DM	1.24 (0.72–2.15)	0.436		
CVA history	1.56 (0.74–3.28)	0.242		
mRS				
3, 4 (control)	1.00	**–**	1.00	–
5	**5.68 (2.85–11.31)**	**<0.001** ^*^	2.73 (0.70–10.66)	0.149
Lesion location				
Cortical stroke involving one hemisphere (control)	1.00	–		
Subcortical stroke involving one hemisphere	0.52 (0.22–1.21)	0.129		
Brainstem + cerebellum stroke (not multifocal)	0.80 (0.34–1.87)	0.607		
Mixed/multifocal stroke	1.15 (0.54–2.44)	0.720		
TOAST classification				
LAA (control)	1.00	–		
Cardioembolic	1.97 (0.98–3.94)	0.055		
Small vessel occlusion	1.31 (0.22–7.82)	0.765		
Other determine	1.31 (0.22–7.82)	0.765		
Undetermined	0.90 (0.31–2.62)	0.850		
NIHSS	1.06 (1.02–1.10)	**0.007** ^*^	0.96 (0.88–1.04)	0.339
GCS ≥ 14	0.33 (0.19–0.58)	**<0.001** ^*^	0.39 (0.12–1.28)	0.121
Hand Brunnstrom (ref: I/II)	0.41 (0.18–0.94)	**0.035** ^*^	1.55 (0.30–7.95)	0.603
Lower limb Brunnstrom (ref: I/II)	0.38 (0.16–0.91)	**0.030** ^*^	1.20 (0.18–8.00)	0.849
ICU stay length	1.08 (1.02–1.15)	**0.009** ^*^	0.96 (0.85–1.09)	0.529
Number of ST sessions during admission	0.73 (0.58–0.92)	**0.008** ^*^	1.29 (0.63–2.63)	0.482

OR, odds ratio; CI, confidence interval; ref, reference; BMI, body mass index; HTN, hypertension; DM, diabetes mellitus; CVA, cerebrovascular accident; mRS, modified Rankin Scale; TOAST, Trial of ORG 10172 in Acute Stroke Treatment; LAA, large-artery atherosclerosis; NIHSS, National Institute of Health Stroke Scale; GCS, Glasgow Coma Scale; ICU, intensive care unit; ST, speech therapy. Model performance indices demonstrated satisfactory fit: Hosmer–Lemeshow test *p* > 0.05; C-statistic AUC > 0.75. *indicates statistical significance (*p* < 0.05). Bold values indicate statistically significant independent variables in the multivariate logistic regression model (*p* < 0.05).

## 4 Discussion

In this retrospective study, cardioembolic stroke was identified as a significant independent predictor of NGT removal failure at 3-months post-discharge. However, given the retrospective design and the exceptionally wide confidence interval, this finding must be interpreted with extreme caution and should be viewed as a preliminary association rather than a definitive risk factor. In addition, stroke location, etiology, severity and patients’ characteristics were all not statistically independent risk factors for NGT removal at discharge. Results emphasized the need to consider cardioembolic stroke in clinical management to improve rehabilitation outcomes.

Although no independent risk factors were identified at discharge for the failure of NGT removal, we believe that this finding could be partially due to the limited sample size. Because nearly all patients with mRS scores of 1 and 2 had their NGT removed at discharge, indicating that mRS scores of 1 and 2 might be an important predictor of successful NGT removal at discharge. Our findings are consistent with the literature, suggesting that low Barthel index (<40) was the independent risk factor for post-stroke dysphagia (16). We believed that the mRS score, or functional status, was crucial for NGT removal, with significant differences evident only in patients with extreme scores. This perfect separation (zero failures) necessitated their exclusion from the multivariable model, representing a highly robust clinical pattern where mild initial disability is a natural predictor of early NGT weaning, though its mathematical modeling is precluded by the lack of failures in this subgroup. We also found that age, sex and stroke-related factors (location, etiology, severity, motor recovery stage) had no significant effects on NGT removal at discharge. This finding is consistent with a previous study (10). We believed that previously mentioned variables likely represent merely one aspect of a stroke. Given the unique nature of each patient, and their functional reserve in response to illness, broader measures like the mRS or the Barthel Index scores could provide a more comprehensive assessment of patients’ disease severity, and their capacity to cope with illness. Consequently, these measures likely better predict the likelihood of successful NGT removal.

As for the risk factors for failure of NGT removal at 3 months after discharge, they involve multiple dimensions, including the intensity of rehabilitation, family and social support, patient motivation, the rate of recovery, and so on. Although these factors are difficult to quantify, or present with objective measures, few studies have ever discussed such a subacute stroke phase. In this study, we applied multivariable regression analysis to identify cardioembolic stroke as a significant independent predictor for the failure of NGT removal at 3 months post-discharge, a finding consistent with multiple studies indicating that cardioembolic stroke is more likely to result in prolonged post-stroke dysphagia (17, 18). This strong correlation is primarily attributed to several distinct pathophysiological and neuroanatomical mechanisms. First, cardioembolic strokes typically feature larger, fibrin-rich thromboemboli of cardiac origin that often occlude proximal major cerebral arteries (such as the internal carotid or middle cerebral artery stem), resulting in extensive, multi-territorial cerebral infarctions, acute large-vessel occlusion (LVO), and higher baseline stroke severity (NIHSS) (19, 20). Second, patients with active cardioembolic sources (such as atrial fibrillation) are highly susceptible to repeated micro-embolism (21). These recurrent, often clinically silent micro-emboli can cause cumulative, multi-territorial damage across distributed swallowing networks (22), thereby severely depleting the brain’s capacity for functional compensation (23). Third, posterior circulation cardioembolism directly threatens the brainstem swallowing centers—specifically the central pattern generators (CPGs) in the medulla (nucleus tractus solitarius and nucleus ambiguus)—as well as the cerebellum, which coordinates swallowing timing and respiratory coupling (24). The profound anatomical impact of these massive lesions severely devastates the widespread, bilaterally distributed cortical and subcortical networks (including the primary motor cortex, insula, and basal ganglia) that collectively govern the complex sensory-motor coordination required for safe swallowing (25). Thus, the synergy of high stroke severity, recurrent micro-embolic load, and direct brainstem/swallowing center involvement collectively explains the profound risk of long-term NGT removal failure in these patients.

Our present findings have important implications for clinical practice in the management and rehabilitation of stroke patients with NGTs. First, patients with cardioembolic stroke must be closely monitored and provided with intensive dysphagia support, as this etiological subtype is clinically associated with more severe initial swallowing impairment and a poorer recovery trajectory (26). Second, since functional status (mRS) is a crucial predictor for successful NGT removal, it is essential to implement an early, comprehensive, interdisciplinary rehabilitation program—comprising not only speech therapy (ST) but also physical therapy (PT) and occupational therapy (OT)—to actively enhance patients’ functional independence during their acute hospital stay (27). Interestingly, our multivariable model indicated that the choice of subsequent post-discharge rehabilitation setting did not significantly affect the likelihood of NGT removal. This finding initially seems to contrast with previous multicenter studies reporting that Taiwan’s intensive Post-Acute Care (PAC) program yields superior functional and tube-weaning outcomes compared to standard inpatient care (28). However, this lack of statistical significance must not be misconstrued as rehabilitation being ineffective. Under Taiwan’s National Health Insurance system, admission to the intensive PAC program is highly selective, strictly requiring patients to exhibit moderate-to-severe functional disability (mRS 3–4) alongside active rehabilitation potential and sufficient physical reserve (29, 30). Conversely, patients discharged to nursing facilities are predominantly those with severe, irreversible impairments (mRS 5) and minimal physiological reserves (31). Because rehabilitation allocation is non-randomized and heavily confounded by baseline neurological severity, the apparent non-significance of the rehabilitation setting in our model highlights a profound selection bias; ultimately, the patient’s baseline physiological reserve remains the dominant determinant of swallowing recovery. The observed discrepancy between studies likely reflects these strict enrollment variations, regional hospital protocols, and distinct patient selection thresholds.

There are several limitations to our study. First, since this study is retrospective design, it limits our ability to account for other factors like nutritional status, gastrointestinal function and family support, which may all influence NGT removal. Second, due to the excessive subcategories of certain items (such as stroke location and Brunnstrom stage) and limited sample size, we used fewer categories to achieve the original classification in the multivariate logistic regression. However, some items had to be excluded due to insufficient sample size to avoid statistically meaningless results. For example, all patients with mRS scores of 1 and 2 had successfully removed their NGTs at 3-months post-discharge (Table 1), but such data were not represented in the final multivariate logistic regression (Table 2). Third, a major limitation of constructing multivariate models in acute stroke cohorts is the intrinsic overlap and high interrelatedness among baseline stroke severity predictors. Scales such as the NIHSS, Glasgow Coma Scale (GCS), and Brunnstrom motor stages all capture different facets of the same underlying neurological deficit. Although we performed multicollinearity diagnostics (using VIF and Tolerance limits) and managed these variables selectively, their clinical and statistical overlap within the regression model inevitably introduces the potential for residual confounding and model instability. This collinearity makes it exceptionally challenging to mathematically isolate the independent effect of any single severity metric, which represents a critical caveat in our regression analysis. Lastly, we did not consider the patients’ pre-stroke functional status. Since everyone’s functional reserve differed, the pre-stroke condition might be one of the main factors influencing the success of NGT removal. In future studies, a prospective design with propensity score matching should be adopted during case enrollment and analysis to further reduce the impact of these interactions on the results.

## 5 Conclusion

In conclusion, this retrospective exploratory analysis identifies critical clinical predictors for NGT removal failure, that functional status (mRS) and stroke etiology may play roles, providing a foundation for a tiered management approach in post-stroke dysphagia care. At discharge, perhaps the lower mRS scores of 1 and 2 were found to be significant predictors for the successful removal of NGT. At 3-months post-discharge, the cardioembolic stroke was the main independent risk factor for the failure in NGT removal, however, these findings represent correlations rather than definitive causal relationships. Our findings offer crucial insights that integrating systematic high-risk screening, early multidisciplinary rehabilitation, and objective-driven weaning assessments may facilitate safer and more efficient NGT management. By flagging high-risk individuals at admission and employing a structured weaning protocol, clinicians can better balance the prevention of premature tube removal against the risks of prolonged dependency.

## Data Availability

The raw data supporting the conclusions of this article will be made available by the authors, without undue reservation.
