# Supplementary material for: Risk factors of nasogastric tube removal failure in acute ischemic stroke patients: a 3-months follow-up cohort study at a single institute
Source: Front Neurol. 2026 Jul 1;17:1801145. doi: 10.3389/fneur.2026.1801145 (PMC13368627; doi:10.3389/fneur.2026.1801145)
Supplement: Supplementary file 1 [file Table_1.DOCX]

**Supplemental Table S1.** Comparison of mRS scores for patients with or without NG tube removal at discharge

| Variables | NG tube removed at discharge | Failure of NG tube removal at discharge |
| --- | --- | --- |
| mRS |  |  |
| 1,2 | 20 (20.8%) | 2 (1.2%) |
| 3,4 | 64 (66.7%) | 78 (47.9%) |
| 5 | 12 (12.5%) | 83 (50.9%) |

*mRS* modified Rankin Scale
